# Invasive breast Cancer treatment in Tanzania: landscape assessment to prepare for implementation of standardized treatment guidelines

**DOI:** 10.1186/s12885-021-08252-2

**Published:** 2021-05-10

**Authors:** Rupali Sood, Nestory Masalu, Roisin M. Connolly, Christina A. Chao, Lucas Faustine, Cosmas Mbulwa, Benjamin O. Anderson, Anne F. Rositch

**Affiliations:** 1 Department of Epidemiology, Johns Hopkins Bloomberg School of Public Health, 615 N. Wolfe St., Office E6150, Baltimore, MD 21205 USA; 2 Bugando Medical Centre, Mwanza, Tanzania; 3 Cancer Research @ UCC, College of Medicine and Health, University College Cork, Cork, Ireland; 4 Breast Health Global Initiative, Fred Hutchinson Cancer Research Center, Seattle, WA 98109 USA

**Keywords:** Breast cancer, Treatment, Low resource, Global oncology, Tanzania

## Abstract

**Background:**

Incidence of breast cancer continues to rise in low- and middle-income countries, with data from the East African country of Tanzania predicting an 82% increase in breast cancer from 2017 to 2030. We aimed to characterize treatment pathways, receipt of therapies, and identify high-value interventions to increase concordance with international guidelines and avert unnecessary breast cancer deaths.

**Methods:**

Primary data were extracted from medical charts of patients presenting to Bugando Medical Center, Tanzania, with breast concerns and suspected to have breast cancer. Clinicopathologic features were summarized with descriptive statistics. A Poisson model was utilized to estimate prevalence ratios for variables predicted to affect receipt of life-saving adjuvant therapies and completion of therapies. International and Tanzanian guidelines were compared to current care patterns in the domains of lymph node evaluation, metastases evaluation, histopathological diagnosis, and receptor testing to yield concordance scores and suggest future areas of focus.

**Results:**

We identified 164 patients treated for suspected breast cancer from April 2015–January 2019. Women were predominantly post-menopausal (43%) and without documented insurance (70%). Those with a confirmed histopathology diagnosis (69%) were 3 times more likely to receive adjuvant therapy (PrR [95% CI]: 3.0 [1.7–5.4]) and those documented to have insurance were 1.8 times more likely to complete adjuvant therapy (1.8 [1.0–3.2]). Out of 164 patients, 4% (*n* = 7) received concordant care based on the four evaluated management domains. The first most common reason for non-concordance was lack of hormone receptor testing as 91% (*n* = 144) of cases did not undergo this testing. The next reason was lack of lymph node evaluation (44% without axillary staging) followed by absence of abdominopelvic imaging in those with symptoms (35%) and lack of histopathological confirmation (31%).

**Conclusions:**

Patient-specific clinical data from Tanzania show limitations of current breast cancer management including axillary staging, receipt of formal diagnosis, lack of predictive biomarker testing, and low rates of adjuvant therapy completion. These findings highlight the need to adapt and adopt interventions to increase concordance with guidelines including improving capacity for pathology, developing complete staging pathways, and ensuring completion of prescribed adjuvant therapies.

**Supplementary Information:**

The online version contains supplementary material available at 10.1186/s12885-021-08252-2.

## Background

Breast cancer is the most common female cancer globally, with most recent estimates of 2.1 million new cases per annum [1]. The incidence of breast cancer continues to rise in low and middle income countries (LMICs), with data from the East African country of Tanzania predicting an 82% increase in breast cancer from 2017 to 2030 [2]. Breast cancer is also the leading cause of cancer-related deaths among females worldwide with 626,679 reported deaths annually, 62.1% of which occur in LMICs [1, 3]. Though mortality rates from breast cancer have decreased in developed countries, breast cancer mortality rates have failed to improve in LMICs or are increasing [1, 4, 5]. While at least 80% of women from high income countries are cured through effective early diagnosis and treatment programs, data from Tanzania show that at least 50% of women diagnosed in this country will die from their breast cancer [2].

Bugando Medical Centre (BMC), a private-public hospital, is one of four tertiary referral hospitals, and one of only three that provides comprehensive cancer care. It serves a population of fourteen million in the Lake Region of Tanzania, where there are very few private facilities that provide diagnostic or treatment services for cancer [2]. Data from the newly established 2016 BMC cancer registry revealed breast cancer to be one of the most burdensome cancers treated at BMC [6]. Most patients reaching BMC for breast cancer treatment are sequentially referred through lower-level health facilities since BMC offers specialty services including diagnostic ultrasound, tissue sampling, pathology/cytology, surgery, medical chemotherapies, endocrine therapies, and recently, radiotherapy [2]. Core oncological staff at BMC include two radiologists trained in breast imaging, two foreign trained medical oncologists, five general surgeons (at least one with specialized training in breast surgery), one radiation oncologist, and two on-site pathologists.

A study conducted in 2012 at BMC reported a 21.8% overall 5 year survival rate for patients with breast cancer; with age at diagnosis, stage of disease, extent of node involvement, and histological grade as significant predictors of overall survival [7]. Another study from Tanzania found late stage of presentation, high proportions of aggressive histologic types of breast cancer, and a general lack of hormone and targeted receptor testing as contributory causes of high mortality in this region [8–10]. Additionally, therapies can be costly and 90% of women in Tanzania remain uninsured due to poor uptake of community-based health initiative schemes to increase insurance coverage for the low socioeconomic rural sector in Tanzania, which may contribute to inequality in access to healthcare [11, 12]. Notably, data on precise medical regimens and the extent of treatment completion, as well as factors that influence treatment adherence, are lacking in this region, as in many LMICs.

To increase survival from breast cancer in Tanzania, it is essential to assess overall capacity to provide systematic breast cancer care. Standardized guidelines provide a framework to treat breast cancer based on key characteristics including lymph node staging, distant metastases evaluation, diagnostic confirmation with histopathology, and hormone receptor testing. In 2017, the National Comprehensive Cancer Network (NCCN) released “harmonized” cancer care guidelines (updated in 2019) specifically for sub-Saharan Africa in collaboration with local oncology centers, which begin to provide guidance on standardized provision of care [13, 14]. Additionally, the Tanzanian Ministry of Health recently released their own national treatment guidelines, including recommendations specific to breast cancer [15–17]. Therefore, we aimed to conduct an in-depth, context-specific assessment of current breast cancer management patterns at the Bugando Medical Centre in order to identify gaps and suggest opportunities to enhance provision of guideline concordant breast cancer care in East Africa and thereby increase survival.

## Methods

### Data source and collection

This is a contemporary, retrospective cohort of patients treated for breast cancer from April 2015–January 2019. Paper charts were acquired by trained BMC research staff from the Oncology, Surgery, Emergency Medicine, and General Medicine departments and cross-referenced with the Tanzania International Association of Cancer Registries database. Data was systematically extracted from the medical charts of patients presenting with breast concerns (*n* = 664) to BMC and suspected to have breast cancer (*n* = 235) (*Supplementary Fig.* *1*). Inclusion criteria for the present analysis of treatment were women ≥30 years of age with either evidence of histopathologic confirmed invasive breast cancer or suggestion of receipt of any form of breast cancer treatment (surgery, chemotherapy, hormone, and/or radiation therapy) based on all available clinical information. As available in the chart, data collected included demographic information (age, sex, date of presentation), sociodemographic data (insurance status, referral possession), clinical characteristics (menopausal status, clinical axillary lymph node status, suspected distant metastases), diagnostic data (diagnostic breast imaging, breast biopsies, breast fine needle aspiration cytology), pathology information (tumor grade, tumor size, nodal evaluation results, breast cancer receptor-status), other staging information (scans), and treatment regimens (surgery, chemotherapy, hormone therapy, radiation therapy).

Age at presentation was calculated using date of birth and date of first presentation to BMC. Paper pathology reports were generally found in the medical charts; however, if these reports were unavailable or missing, but pathology was mentioned in the clinical notes, all efforts were made to use a simplified electronic database to retrospectively find filed pathology reports with aid of medical staff. Histopathology report turnaround time was then calculated from the day of surgery or biopsy to the day of report receival by the designated treatment team. For analytic purposes, chemotherapy regimens were classified in four groups: anthracycline and taxane-based (adriamycin, cyclophosphamide, taxol [AC-T]), anthracycline alone (cyclophosphamide, adriamycin, 5-fluorouracil [CAF]), taxane alone, and other (gemcitabine, capecitabine, or unknown). “Referral” was defined as documented referral from a lower-level facility to BMC either as a physical referral form or mention in clinical notes. Clinically positive nodes were defined where documentation was present of palpable axillary lymphadenopathy with or without targeted breast imaging. Suspected distant metastases was defined as imaging suspicious for distant disease (pulmonary, abdominal, or skeletal). At the time of the study, immunohistochemistry (IHC) was not always available on site and samples had to be outsourced to India for hormone testing at the patients’ expense [18]. Estrogen receptor (ER) and progesterone receptor (PR) positivity were defined as nuclear labeling ≥1% by IHC and human epidermal growth factor receptor 2 (HER2) positivity was defined as an IHC score of 3 + .

To increase accuracy, all data was correlated with previously collected clinical data from a large ongoing review of patients seeking care for breast concerns at BMC. Clinical notes from charts were used to supplement report data. Local staff and physicians were consulted to resolve any discrepancies identified during data collection. This study was approved by the Institutional Review Boards at Johns Hopkins Bloomberg School of Public Health, the Catholic University of Health and Allied Sciences, and the Tanzanian National Institute for Medical Research.

### Statistical analysis

All demographic information and clinicopathologic features were summarized with the use of descriptive statistics. Patients were grouped into analytic subsets based on presence of confirmed histopathology as a basis for further oncological care. Chi-square (χ2) tests were used to test for significance of associations between categorical variables. Given the high prevalence of the chosen outcomes of adjuvant therapy receipt (chemotherapy, hormone therapy, radiation therapy) and completion of prescribed adjuvant chemotherapy, Poisson regression was used to estimate univariate and multivariate prevalence ratios (PrRs). Covariates included median age, documented referral to BMC, Mwanza city residence, insurance status, histopathologic confirmed breast cancer, clinically positive nodes, and suspected distant metastases. Goodness-of-fit parameters were estimated, and variance inflation factors were inspected to ensure no collinearity between covariates in the model. Survival was not modelled since post-treatment surveillance was lacking for most cases. All analyses were performed using Stata v.15. Statistically significance was defined as a *p*-value < 0.05.

We assessed whether breast cancer care pathways at BMC were guideline concordant with NCCN Harmonized Guidelines™ for Sub-Saharan Africa and Tanzania specific guidelines in the domains of lymph node evaluation (fine needle aspiration [FNA] or axillary nodal dissection during surgery), distant staging (abdominal imaging with or without chest imaging), confirmed histopathology diagnosis, and receptor testing (ER and/or PR) [13, 16]. A “concordance” score of 0–4 was calculated for each individual case based on these domains.

## Results

### Clinicopathologic characteristics and management of breast Cancer cases

A total of 164 patients who received any treatment for breast cancer were eligible for inclusion *(*Table 1*, Supplementary Fig.* *1**).* The median age was 50 years (range: 30–93 years). Approximately half of the women (45%) were noted to possess a referral from a lower tier hospital to BMC, and the majority were from the Mwanza region (61%). Most patients (70%) did not have documented insurance. A majority of the women were post-menopausal (43%), while 37% were pre-menopausal (20% status not reported). All women presented with one or more breast symptoms (37% with a palpable lump, 26% with pain, 69% with swelling). A total of 14% (23/164) of women were documented as having clinically positive nodes, while 16% (26/164) of women were suspected to have distant metastatic disease. Most women underwent some form of surgery (82%) with unilateral mastectomy being the most frequent type (65%). Only 56% (*n* = 75) of the women who underwent surgery also received axillary evaluation in the form of dissection. Chemotherapy was prescribed to 63% of these women but was based on histopathologic confirmed breast cancer in only 56% of the cases. Almost 80% of women prescribed chemotherapy were documented as having received at least one cycle of their chemotherapy regimen. Hormone therapy, either tamoxifen or anastrozole for a total of 5 years, was prescribed to 38% (63/164) of the women, although only 9 of these patients had positive hormone receptor testing results. A total of 3% (5/164) of women received radiation therapy although close to 20% of patients were treated with partial mastectomy (lumpectomy) *(*Table 1*)*.

**Table 1:** Characteristics and Clinical Management of all Patients treated for Breast Cancer.

Characteristic (***n*** = 164)	Number (%)
**Median age at presentation, range (*n*** **= 164)**	50, 30–93 years
**Referral (*n*** **= 164)**^a^	
Yes	74 (45%)
No	90 (55%)
**Mwanza City Residence (*n*** **= 164)**^b^	
Yes	34 (21%)
No	130 (79%)
**Insurance (*n*** **= 164)**	
Yes	50 (30%)
No	114 (70%)
**Menopause Status (*n*** **= 164)**	
Pre-menopausal	61 (37%)
Post-menopausal	70 (43%)
Not reported	33 (20%)
**Breast Symptoms at Presentation (*n*** **= 164)**	
Yes	164 (100%)
No	0 (0%)
**Clinically Positive Nodes Reported (*n*** **= 164)**	
Suspected	23 (14%)
Not suspected^c^	141 (86%)
**Suspected Distant Metastases (*n*** **= 164)**	
Yes	26 (16%)
No	138 (84%)
**Surgery (*n*** **= 164)**	
Yes	135 (82%)
No	29 (18%)
**Surgical Procedure (*n*** **= 135)**	
Excision/Lumpectomy	25 (19%)
Unilateral Mastectomy	88 (65%)
Bilateral Mastectomy	4 (3%)
Other/Unknown	18 (13%)
**Axillary evaluation (*n*** **= 135)**	
Yes	75 (56%)
No	60 (44%)
**Chemotherapy prescribed (*n*** **= 164)**	
Yes	103 (63%)
No	61 (37%)
**Chemotherapy basis (*n*** **= 103)**	
Proven histopathology	57 (56%)
Symptoms	2 (2%)
Other (imaging, FNAC, clinical suspicion)	43 (42%)
**Chemotherapy Received (*n*** **= 103)**	
Yes	81 (79%)
No	22 (21%)
**Hormone therapy (*n*** **= 164)**	
Yes	63 (38%)
No	101 (62%)
**Basis for hormone therapy (*n*** **= 63)**	
Proven receptor status	9 (15%)
Clinical suspicion	54 (86%)
**Radiation therapy (*n*** **= 164)**	
Yes	5 (3%)
No	197 (97%)
**Adjuvant therapy**^**d**^	
Yes	100 (61%)
Chemotherapy	81 (81%)
No	64 (39%)

^a^ Defined as referral slip present in chart or mention of referral in clinical notes, ^b^ Defined as residence in districts of Nyamagana and Ilemela. ^c^ Includes those with no documented clinical axillary exam recorded, ^d^ Includes chemotherapy, hormone therapy, and radiation therapy

*FNAC*: fine needle aspiration cytology

Overall, 113 (69%) cases had histopathological confirmation of breast cancer with pathology reports available for review *(*Table 2*, Supplementary Fig.* *1**).* Of these, 95% of the reports were from surgical sampling or excisions, while 5% were from needle biopsies prior to surgery. All were invasive carcinomas. The median turnaround time (range) for histopathology reports was 36 days (range 1–202 days); 64% were returned to the treating physician in > 1 month. Regarding distant cancers staging, scans for those with confirmed breast cancer included chest x-rays (*n* = 10), chest computed tomography (CT) scans (*n* = 26), abdominal ultrasounds (*n* = 48), and abdominal CT scans (*n* = 18). Final nodal status, based on pathology results from surgical evaluation with axillary node dissections, was positive in 34% of the cases, negative in 9% of the cases (57% not reported). Regarding tumor size, 3% were < 2 cm, 24% were 2–5 cm, 26% were > 5 cm and 47% were not reported. The greatest fractions of carcinomas were Nottingham grade III (34%), while 30% were grade II, 3% were grade I and 33% were not reported. Regarding hormone receptor profiling, ER and PR testing were performed in 10 (9%) of cases; 4 ER−/PR-, 5 ER+/PR+, and 1 ER+/PR-. All with hormone receptor positive disease received some form of hormone therapy. HER2 receptor status was tested by IHC in 7 (6%) of patients; 3 HER2+ and 4 HER2-. No targeted HER2 therapy was provided.

**Table 2:** Clinicopathologic Characteristics of Histopathology Confirmed Treated Breast Cancer Cases.

Histopathology Confirmed Cases (***n*** = 113) Clinicopathologic Characteristic	Number (%)
**Histopathology Method (*n*** **= 113)**	
Core Biopsy	16 (14%)
Surgical Sample	97 (86%)
**Histopathology Report Time (*n*** **= 107**^**)a**^	
Median (range, days)	36 (1–202)
< 1 month	39 (36%)
> 1 month	68 (64%)
**Treatment Staging: Chest (*n*** **= 113)**	
Chest X-Ray	10 (9%)
Chest CT^b^	29 (26%)
None	74 (65%)
**Treatment Staging: Abdomen (*n*** **= 113)**	
Abdominal ultrasound	48 (42%)
Abdominal CT scan^c^	27 (24%)
None	38 (34%)
**Nodal Status (*n*** **= 113)**	
Positive	38 (34%)
Negative	10 (9%)
Not reported	65 (57%)
**Tumor Size (*n*** **= 113)**	
< 2 cm	3 (3%)
2–5 cm	27 (24%)
> 5 cm	30 (26%)
Not reported	53 (47%)
**Tumor Grade (*n*** **= 113)**	
Grade I	3 (3%)
Grade II	34 (30%)
Grade III	39 (34%)
Not reported	37 (33%)
**Receptor Status (*n*** **= 113)**	
Hormone Receptor Tested	10 (9%)
ER/PR negative	4 (40%)
ER/PR positive	5 (50%)
ER positive/PR negative	1 (10%)
Hormone Receptor Not tested	103 (91%)
HER2 Receptor Tested	7 (6%)
Positive	3 (43%)
Negative	4 (57%)
HER2 Receptor Not tested	106 (94%)

^a^ Six cases without reported histopathology report turnaround time, ^b^ Computed tomography includes those who received both X-Ray and CT, ^c^ Includes those who received both ultrasound and CT

*CT:* Computed Tomography; *ER*: Estrogen Receptor; *PR*: Progesterone Receptor; *HER2:* Human Epidermal Growth Factor Receptor 2

### Breast Cancer pathway guideline concordance

The four overlapping domains of concordance for breast cancer management from the NCCN Harmonized and Tanzania Ministry of Health guidelines were lymph node evaluation, distant staging evaluation with imaging for patients presenting with any symptoms, histopathological diagnosis, and hormone receptor testing (Table 3). Out of 164 patients, 4% (*n* = 7) received concordant care based on evaluation of all four management domains, while 24% (*n* = 39) met concordance in three domains, 35% (*n* = 58) met concordance in two domains, and 29% (*n* = 47) met concordance in one domain. A total of 8% (*n* = 13) underwent work-up that was non-concordant in all domains. The first most common reason for non-concordance was lack of hormone receptor testing as 91% (144/164) of cases did not undergo hormone receptor testing. The next most common reason for non-concordance was lack of lymph node evaluation (44% without axillary staging) followed by absence of abdominal pelvic imaging (35%) and then lack of histopathological confirmation (31%).

**Table 3:** NCCN and Tanzanian Guidelines Based Opportunities for Improvement.

Category	NCCN Harmonized Guideline	Tanzania Guideline	Data from Bugando Medical Centre	Recommendation
**Lymph Node Evaluation**	Node evaluation should be performed minimally with a full axillary lymph node dissection.	Before intervention, an attempt should be made to stage all patients using proper TNM parameters.	• 75/164 (56%) received axillary staging • 60/164 (44%) no axillary staging	Axillary evaluation for all patients
**Staging Evaluation**	If symptomatic, chest imaging (x-ray/CT) and abdominal imaging (ultrasound/CT) should be performed.	Chest x-ray and CT chest with contrast if pulmonary symptoms, abdominal pelvic US should always be performed.	• All treated breast cancer patients presented with focal breast symptoms • Abdominal pelvic US in 107/164 (65%) of patients • Only 37/164 (23%) underwent both chest and abdominal imaging	Abdominal US for all patients given high burden of late-stage disease
**Histopathology**	Cancer diagnosis should be confirmed with histopathology.	Histopathology should be reported by specialist pathologists, and reviewed with a panel of pathologists before treatment is instituted at a specialist treatment center.	• 113/164 (69%) histopathology confirmed cases • 51/164 (31%) treated on the basis of clinical suspicion	Confirmation histopathology for diagnosis in all patients
**Hormone Receptor Testing**	Hormone receptor testing should be performed to subtype cancer and guide treatment.	Immunohistochemistry for ER and PR must be done.	• 13/164 (9%) underwent ER/PR testing • 144/164 (91%) did not undergo receptor testing	ER/PR receptor testing at for all patients to select patients for adjuvant endocrine therapy

*TNM*: Tumor, Node, Metastases; *CT:* Computed Tomography; *ER*: Estrogen Receptor; *PR*: Progesterone Receptor

### Chemotherapy prescription and receipt

**Fig. 1:**
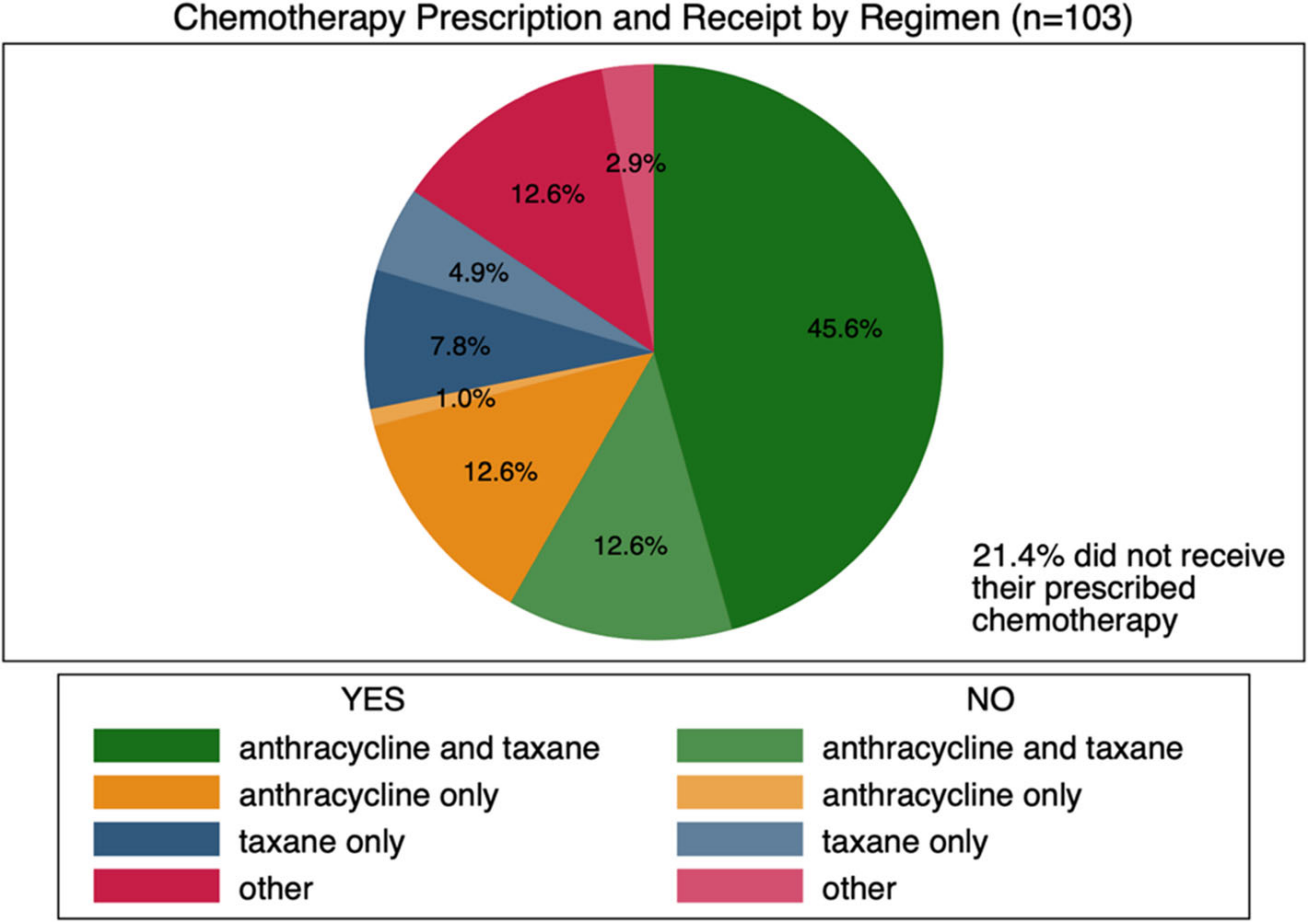
Chemotherapy Receipt for all 103 Breast Cancer Cases Prescribed Chemotherapy by type of Regimen. Yes = some portion of chemotherapy received, No = no portion of chemotherapy received

Chemotherapy was documented as being prescribed for 63% (103/164) of patients. Of these, 79% (*n* = 81) were reported to have received at least one cycle of their chemotherapy regimen *(*Fig. 1*)*, while only 51% (*n* = 53) patients completed their prescribed regimens. Prescribed chemotherapy included anthracycline and taxane-based (AC-T) and anthracycline only (CAF) regimens, and taxane only regimens. These were given parenterally every 3–4 weeks for 6 cycles. When metastatic disease was suspected, gemcitabine or capecitabine, categorized as other, were often prescribed. The most common adjuvant regimens were anthracycline and taxane-based, prescribed to 60 patients (58%); 45.8% of those prescribed the regimen took some portion of it. Based on prescribing patterns and chemotherapy receipt, 12.6% received anthracycline only (13.6% prescribed), 7.8% received taxane only (12.6% prescribed), and 12.8% received other (15.5% prescribed). A total of 21.4% of patients did not receive any portion of their prescribed regimen *(*Fig. 1*).*

### Adjuvant therapy receipt and adjuvant chemotherapy completion

Of 164 patients, 100 (61%) patients received some form of adjuvant therapy (chemotherapy, endocrine therapy and/or radiation), while 64 (39%) did not. There was no difference in prevalence of adjuvant therapy receipt in univariable analyses (PrR [95%CI]) based on age < or ≥ median age of 50 years (1.0 [0.7–1.5]), referral (0.9 [0.6–1.3]), Mwanza city residence (1.0 [0.7–1.6]), clinically positive nodes (1.1 [0.6–1.9]) or suspected distant metastases (1.1 [0.7–1.8]). Insurance status (1.5 [1.0–2.2]) yielded a borderline difference in prevalence of adjuvant therapy receipt *(*Table 4*).* These relationships were maintained in multivariable analysis. However, in both univariable and multivariable analyses, patients with histopathologic confirmation of breast cancer were 3 times as likely to receive adjuvant therapy than those who did not have a formal histopathological diagnosis (3.0 [1.7–5.4]).

**Table 4:** Univariable and Multivariable Analysis of Factors Associated with Prevalence of Treatment

	Adjuvant Therapy Receipt (***n*** = 164)^**a**^		Completion of Prescribed Adjuvant Chemotherapy (***n*** = 81)^**b**^	
**Data Variable**	Univariable	Multivariable	Univariable	Multivariable
	PrR^a^ (95% CI)	PrR (95% CI)	PrR (95% CI)	PrR (95% CI)
Age^a^				
< 50 years	REF	REF	REF	REF
≥ 50 years	1.0 (0.7–1.5)	1.0 (0.7–1.5)	0.8 (0.5–1.5)	0.9 (0.5–1.6)
Referral^b^	0.9 (0.6–1.3)	0.9 (0.6–1.3)	0.9 (0.5–1.6)	0.8 (0.5–1.6)
Mwanza City^c^	1.0 (0.7–1.8)	1.0 (0.6–1.7)	1.0 (0.5–2.1)	1.1 (0.6–2.1)
Insurance	1.5 (1.0–2.2)	1.3 (0.9–2.0)	1.8 (1.0–3.2)	1.7 (0.9–3.1)
Histopathological Diagnosis^d^	3.0 (1.7–5.4)	3.0 (1.7–5.4)	1.1 (0.4–2.7)	1.4 (0.5–3.5)
Clinically Positive Nodes^e^	1.1 (0.6–1.9)	1.0 (0.6–1.9)	1.6 (0.8–3.1)	1.4 (0.7–2.9)
Suspected Distant Metastases^f^	1.1 (0.7–1.8)	1.2 (0.7–2.2)	1.8 (1.0–3.4)	1.5 (0.7–3.3)

^a^Categorized based on median age, ^b^Chart documented referral (slip or mention), ^c^residence in vs. out of Mwanza city, ^d^As documented in the chart, ^e^Documentation of palpable axillary lymphadenopathy with or without targeted breast imaging, ^f^Imaging suspicious for distant disease (pulmonary/abdominal/skeletal)

*PrR* prevalence ratio

Overall, 103 patients were prescribed chemotherapy and 81 of these were in the adjuvant setting with only 48 (59%) of patients completing their prescribed adjuvant chemotherapy, while 33 (41%) did not. There was no significant difference in the prevalence of adjuvant chemotherapy completion in univariable analyses (PrR [95% CI]) based on median age (0.8 [0.5–1.5]), referral (0.9 [0.5–1.6]), Mwanza city residence (1.0 [0.6–1.9]), histopathological diagnosis (1.1 [0.4–2.7]), and clinically positive nodes, or suspected distant metastases *(*Table 4*).* However, those documented to have insurance were 1.8 times more likely to complete adjuvant chemotherapy versus those who did not have documented insurance (1.8 [1.0–3.2]). Multivariable analyses did not reveal any additional statistically significant relationships.

## Discussion

Given the global challenges of late-stage presentation and a lack of advanced infrastructure in many areas of sub-Saharan Africa and other LMICs, zonal hospitals (tertiary facilities) are tasked with providing oncological care within severely resource-constrained parameters [13, 17, 19]. Little is known about true cancer care patterns in East Africa, so we aimed to assess the current landscape of breast cancer management to identify opportunities for achieving guideline-concordant care based on contextually appropriate national and international guidelines [13, 16]. In a comprehensive medical chart review of over 150 breast cancer patients at one of the only cancer hospitals in northern Tanzania, we found that 91% of patients with breast cancer did not undergo hormone receptor testing, 44% did not receive axillary evaluation, 35% of those with symptoms did not undergo abdominal evaluation for metastatic disease, and 31% of cases were not confirmed by histopathology. Our analyses showed that having a histopathologic diagnosis increased the likelihood of receiving adjuvant therapy and that those with insurance were more likely to complete their prescribed adjuvant chemotherapy regimen. Based on these findings, we suggest implementation of key high-value clinical interventions with proven therapeutic benefits that should be systematically adapted and adopted to prevent unnecessary deaths from breast cancer. Specifically, these include the development and enactment of standardized patient pathways to facilitate complete oncologic staging, strengthening of pathology/receptor testing capacity, and provision of resources to enhance completion of prescribed adjuvant therapy.

Administration of evidence-based oncological therapies are most effective when they are provided based on disease extent and receptor status [13, 16]. Our data show that though surgical staging depends on tumor size, lymph node status, and metastases evaluation, 79% of patients received surgery, but axillary nodal evaluation results were only available for 56% of patients. Previous data from BMC has also shown that although there are five general surgeons on staff at Bugando, there are no standardized institutional guidelines in regards to axillary node clearance [12]. Additionally, though all patients presented with symptoms of locally advanced disease, testing for distant metastases was infrequent [13, 20]. This is consistent with previous studies, including data from 2016 revealing that 77.8% of cases captured in the BMC breast cancer registry lacked complete staging information [6]. It has long been known that axillary evaluation portends a survival benefit with the earliest randomized study demonstrating an 18.6% failure rate within 2 years for the women who were randomized to only receive mastectomy without axillary lymph node evaluation [21]. Although sentinel node lymph node biopsy has been shown to be noninferior to axillary lymph node dissection and less invasive, it is lacking as a surgical technique in this area [20]. As comprehensive staging is vital not only for disease extent classification, but for guiding data-driven interventions with a direct link to a survival benefit, the importance of enhancing staging evaluation and documentation cannot be overstated.

We additionally found that having a confirmed histopathologic diagnosis was associated with an increased likelihood of receiving subsequent adjuvant therapy, but only 113 of cases were confirmed by histopathology (69%). One known issue is pathology capacity. In Tanzania, there only 22 pathologists in the public sector serving a population of 48 million, although BMC is fortunate to have two full time pathologists [2, 22]. Cost may also contribute to the relationship between histopathological diagnosis and adjuvant therapy completion as affording out-of-pocket payments for histopathology may serve as a proxy for having the means to afford subsequent adjuvant therapies. Long report turnaround times, which ranged from 1 to 202 days (median: 36 days) may also mean that some women are lost to follow-up, especially since many women must travel from surrounding villages to BMC for therapies.

When pathology reports were available, our data showed ER/PR receptor testing for only 15% of women and HER2 receptor testing for 6% of patients. Given that hormone therapy is the one medication that can be prescribed and taken by a patient without having to return for cyclic visits or as frequent monitoring, improving receptor testing would be an extremely high-yield intervention to address. Due to the lack of on-site testing availability at the time of the study, mostly those who were able to afford costs of outsourced testing, which was being performed in India (external laboratory) received receptor status information. It is known that patients generally incur high out of pocket expenditures for receptor testing, pathology diagnosis, and diagnostic imaging/procedures, which makes cancer sub-typing difficult and treatment patterns thus more unpredictable [2, 18]. Therefore, interventions to achieve guideline concordant care must target both institutional capacity for pathology—which is currently ongoing at BMC--and patient-level barriers, including affordability, so that patient with all cancer types can then receive optimal care.

This essential care encompasses all medical oncologic therapies (chemotherapy, hormone therapy, targeted therapy, radiation therapy), which provide most benefit when affordable, accessible, and administered to completion based on cancer subtype. BMC offers most therapies on the World Health Organization Model List of Essential Medicines, as well as offering access to radiation therapy [23]. Though it is known that lumpectomy plus radiation therapy provides the same if not better survival benefit than mastectomy, our data showed that close to 20% of patients were treated with excision/lumpectomy, but only five (3%) women also received radiation therapy [24]. As radiation therapy is one of the newer available therapies at BMC, clinical care pathway improvements should include a focus on strengthening radiation therapy provision alongside lumpectomy therapy.

Chemotherapy regimens can only offer maximal benefit when properly sequentially administered in multi-agent regimens that have proven to be efficacious. We found that while chemotherapy (neo-adjuvant and adjuvant) was commonly prescribed (63% of all patients), and 79% (*n* = 81) of patients were reported to have received at least one cycle of their chemotherapy regimen*,* only 51% (*n* = 53) of patients with a confirmed histopathological diagnosis completed their prescribed regimens [23]. Receipt of one or even a few doses of chemotherapy does not provide a survival benefit, uses up resources that may already be scarce, and may cause toxicity for the patient without achieving the intended therapeutic benefit. The lack of chemotherapy completion also suggests challenges with the affordability and repeat access to care as 21% of all patients prescribed a chemotherapy regimen did not complete any portion of their regimens (Fig. 1) [7]. Our model showed health insurance, a factor known to influence accessibility and affordability of care, to be associated with the completion of adjuvant chemotherapy [25]. Despite the National Health Insurance Fund becoming available in 2001 in Tanzania, 90% of women remain uninsured. At BMC, free of cost therapies are often in short supply so women must purchase most of their own medications [12]. Other recognized reasons for non-completion of chemotherapy regimens, which may not have been captured in our study, include tolerability of chemotherapy toxicities, relative feeling of well-being after initial chemotherapy doses, seeking alternative treatments due to drug side effects, and increased travel time as cycles of adjuvant chemotherapy require repeated visits [26–28]. Decreasing out of pocket costs for medical therapies, by increasing insurance coverage, and by providing local hostel-type accommodations, which is already being done for pediatric oncology patients, could enhance completion of breast cancer treatment regimens, which is directly linked to decreased mortality [25, 29, 30].

One limitation of this study is the potential for unreported/unrecorded data in clinical charts, which may lead to an inaccurate representation or under-estimate provision of diagnostic testing or treatment. Though all efforts were made to find data, including official pathology reports, missing data is a limitation of paper-based records, one that also has potential clinical implications on subsequent provision of optimal clinical care. Fortunately, BMC has recently transitioned to a hospital-wide electronic medical record system which is expected to enhance record keeping, specifically for breast cancer patients, and allow for facilitation of information sharing across departments [30]. The smaller sample size is also a limitation as the study may be under-powered to detect differences in care by various patient or clinical characteristics. Unlike recent studies focusing only on initiation of adjuvant therapies, our study provides a more in-depth, patient specific analysis of actual diagnostic pathways and treatment patterns including completion of chemotherapy [31]. Overall survival could not be measured directly since many patients’ last visit to the zonal hospital in Mwanza was on their last day of treatment. However, given its strong association with decreased mortality and lower risk of recurrence, adjuvant chemotherapy completion was used as a clinically relevant endpoint in the current study, acknowledging the possibility, although very unlikely in the Lake Region, that a proportion of continued chemotherapy could have been received at another facility [2, 13, 32, 33]. Despite these inherent limitations in the available data, the assessment process and findings from this study can be used to inform improvements in breast cancer care for future patients, including ongoing interventions as part of a larger organizational study in the region [34]. Directly engaging the cancer patient population will be a critical step to ensuring that patient-reported barriers to care are also addressed and interventions are appropriately adapted and acceptable [35, 36]. Additionally, while providers at BMC are internationally trained, they may not be intimately familiar with the NCCN and Tanzania specific guidelines as these are quite new [30]. Dissemination of these standardized guidelines will likely take time and may necessitate supplemental training offerings, the impact of which can be evaluated in future studies.

## Conclusions

In conclusion, our study indicates a strong foundation for the multidisciplinary treatment of breast cancer at the BMC with key areas for improvement. Patient-specific clinical data from Tanzania show limitations of current breast cancer management including axillary staging, receipt of formal diagnosis, lack of predictive biomarker testing, and low rates of adjuvant therapy completion. Key interventions to undertake to increase survival for breast cancer patients are: improving capacity to meet patient-volume demands for pathology and hormone receptor testing, developing pathways for complete surgical staging (additional imaging, lymph node evaluation), and providing access to/ensuring completion of prescribed adjuvant chemotherapies. Future research may focus on the effectiveness of interventions informed by these clinical data in improving outcomes for breast cancer patients treated at BMC, which will serve as a first step in upscaling capacity to meet standardized treatment guidelines and improve patient care.

## Supplementary Information

**Additional file 1 Supplementary Fig. 1.** Eligibility, inclusion, and exclusion criteria of study population: Among 235 patients with suspected breast reviewed during the study period, 164 patients were included.

## Data Availability

The datasets used and/or analyzed during the current study are available from the corresponding author on reasonable request.

## Abbreviations

LMIC: Low- and Middle-Income Countries
BMC: Bugando Medical Centre
NCCN: National Comprehensive Cancer Network
ER: Estrogen Receptor
PR: Progesterone Receptor
IHC: Immunohistochemistry
HER2: Human Epidermal Growth Factor Receptor
AC-T: Adriamycin, Cyclophosphamide, Taxol
CAF: Cyclophosphamide, Adriamycin, 5-Fluorouracil
PrR: Prevalence Ratio
FNA: Fine Needle Aspiration
CT: Computed Tomography
CI: Confidence Interval

## References

[1] Bray F, Ferlay J, Soerjomataram I, Siegel RL, Torre LA, Jemal A (2018). Global cancer statistics 2018: GLOBOCAN estimates of incidence and mortality worldwide for 36 cancers in 185 countries. CA Cancer J Clin.

[2] Bishop A, Duggan C, Dvaladze A: Tanzania Breast Health Care Assessment 2017: An assessment of breast Cancer early detection, Diagnosis, and Treatment in Tanzania. 2017.

[3] Torre LA, Bray F, Siegel RL, Ferlay J, Lortet-Tieulent J, Jemal A (2015). Global cancer statistics, 2012. CA Cancer J Clin.

[4] Torre LA, Siegel RL, Ward EM, Jemal A (2016). Global Cancer incidence and mortality rates and trends--an update. Cancer Epidemiol Biomark Prev.

[5] Ginsburg O, Rositch AF, Conteh L, Mutebi M, Paskett ED, Subramanian SJCBCR. Breast cancer disparities among women in low- and middle-income countries. 2018;10(3):179–86.

[6] Olson AC, Afyusisye F, Egger J, Noyd D, Likonda B, Masalu N, Suneja G, Chao N, Zullig LL, Schroeder K (2020). Cancer incidence and treatment utilization patterns at a regional cancer center in Tanzania from 2008-2016: initial report of 2,772 cases. Cancer Epidemiol.

[7] Mabula JB, McHembe MD, Chalya PL, Giiti G, Chandika AB, Rambau P, Masalu N, Gilyomai JM (2012). Stage at diagnosis, clinicopathological and treatment patterns of breast cancer at Bugando medical Centre in North-Western Tanzania. Tanzan J Health Res.

[8] Burson AM, Soliman AS, Ngoma TA, Mwaiselage J, Ogweyo P, Eissa MS, Dey S, Merajver SD (2010). Clinical and epidemiologic profile of breast cancer in Tanzania. Breast Dis.

[9] Mwakigonja AR, Lushina NE, Mwanga A (2017). Characterization of hormonal receptors and human epidermal growth factor receptor-2 in tissues of women with breast cancer at Muhimbili National Hospital, Dar es salaam, Tanzania. Infect Agent Cancer.

[10] Mbonde MP, Amir H, Schwartz-Albiez R, Akslen LA, Kitinya JN (2000). Expression of estrogen and progesterone receptors in carcinomas of the female breast in Tanzania. Oncol Rep.

[11] Ajuaye A, Verbrugge B, Van Ongevalle J, Develtere P (2019). Understanding the limitations of "quasi-mandatory" approaches to enrolment in community-based health insurance: empirical evidence from Tanzania. Int J Health Plann Manag.

[12] Bintabara D, Nakamura K, Seino K (2018). Improving access to healthcare for women in Tanzania by addressing socioeconomic determinants and health insurance: a population-based cross-sectional survey. BMJ Open.

[13] Gradishar WJ, Anderson BO, et. NCCN Harmonized Guidelines for Sub-Saharan Africa: Breast Cancer. 2019. [https://www.nccn.org/professionals/physician_gls/pdf/breast_harmonized-africa.pdf].

[14] Anderson BO (2020). NCCN harmonized guidelines for sub-Saharan Africa: a collaborative methodology for translating resource-adapted guidelines into actionable in-country Cancer control plans. JCO Glob Oncol.

[15] Buguzi S (2018). Guidelines on cancer treatment coming. In: The Citizen.

[16] The Ministry of Health CD, Gender, Elderly, and Children National Guidelines for Cancer Management. 2018.

[17] DeBoer RJ, Ndumbalo J, Meena S, Ngoma MT, Mvungi N, Siu S, Selekwa M, Nyagabona SK, Luhar R, Buckle G (2020). Development of a theory-driven implementation strategy for cancer management guidelines in sub-Saharan Africa. Implement Sci Commun.

[18] Bollyky TJ, Templin T, Cohen M, Dieleman JL (2017). Lower-income countries that face the Most rapid shift in noncommunicable disease burden are also the least prepared. Health Aff (Millwood).

[19] Kene TS, Odigie VI, Yusufu LM, Yusuf BO, Shehu SM, Kase JT (2010). Pattern of presentation and survival of breast cancer in a teaching hospital in North Western Nigeria. Oman Med J.

[20] Giuliano AE, Connolly JL, Edge SB, Mittendorf EA, Rugo HS, Solin LJ, Weaver DL, Winchester DJ, Hortobagyi GN (2017). Breast Cancer-major changes in the American joint committee on Cancer eighth edition cancer staging manual. CA Cancer J Clin.

[21] Fisher B, Anderson S, Bryant J, Margolese RG, Deutsch M, Fisher ER, Jeong JH, Wolmark N (2002). Twenty-year follow-up of a randomized trial comparing total mastectomy, lumpectomy, and lumpectomy plus irradiation for the treatment of invasive breast cancer. N Engl J Med.

[22] Sayed S, Lukande R, Fleming KA (2015). Providing pathology support in low-income countries. J Glob Oncol.

[23] Organization WH: World Health Organization Model List of Essential Medicines. In*.* Edited by Organization WH, 21st edn; 2019.

[24] Hwang ES, Lichtensztajn DY, Gomez SL, Fowble B, Clarke CA (2013). Survival after lumpectomy and mastectomy for early stage invasive breast cancer: the effect of age and hormone receptor status. Cancer.

[25] Hsia J, Kemper E, Sofaer S, Bowen D, Kiefe CI, Zapka J, Mason E, Lillington L, Limacher M (2000). Is insurance a more important determinant of healthcare access than perceived health? Evidence from the Women's Health Initiative. J Womens Health Gend Based Med.

[26] Adisa AO, Lawal OO, Adesunkanmi AR (2008). Paradox of wellness and nonadherence among Nigerian women on breast cancer chemotherapy. J Cancer Res Ther.

[27] Dickens C, Joffe M, Jacobson J, Venter F, Schuz J, Cubasch H, McCormack V (2014). Stage at breast cancer diagnosis and distance from diagnostic hospital in a periurban setting: a south African public hospital case series of over 1,000 women. Int J Cancer.

[28] Goudge J, Gilson L, Russell S, Gumede T, Mills A (2009). Affordability, availability and acceptability barriers to health care for the chronically ill: longitudinal case studies from South Africa. BMC Health Serv Res.

[29] Kersten E, Scanlan P, Dubois SG, Matthay KK (2013). Current treatment and outcome for childhood acute leukemia in Tanzania. Pediatr Blood Cancer.

[30] Amadori D, Serra P, Bucchi L, Altini M, Majinge C, Kahima J, Botteghi M, John C, Stefan DC, Masalu N (2016). The Mwanza Cancer project. Lancet Oncology.

[31] Foerster M, Anderson BO, McKenzie F, Galukande M, Anele A, Adisa C, Zietsman A, Schuz J, Dos Santos SI, McCormack V (2019). Inequities in breast cancer treatment in sub-Saharan Africa: findings from a prospective multi-country observational study. Breast Cancer Res.

[32] Ho PJ, Ow SGW, Sim Y, Liu J, Lim SH, Tan EY, Tan SM, Lee SC, Tan VK, Yap YS (2020). Impact of deviation from guideline recommended treatment on breast cancer survival in Asia. Sci Rep.

[33] Early Breast Cancer Trialists' Collaborative G (2018). Long-term outcomes for neoadjuvant versus adjuvant chemotherapy in early breast cancer: meta-analysis of individual patient data from ten randomised trials. Lancet Oncol.

[34] Rositch AF, Chao C, Passaniti A, Mwakatobe K, Visvanathan K, Masalu N. Mixed-Methods Evaluation of Multiple Perspectives on Breast Cancer Control to Guide Stakeholder Selection of Implementation Strategies: The Time to A.C.T. Study in Mwanza, Tanzania. J Glob Oncol. 2020;2020(NCl1):43.

[35] Rositch AF, Unger-Saldana K, DeBoer RJ, Ng'ang'a A, Weiner BJ (2020). The role of dissemination and implementation science in global breast cancer control programs: frameworks, methods, and examples. Cancer.

[36] Mutebi M, Anderson BO, Duggan C, Adebamowo C, Agarwal G, Ali Z, Bird P, Bourque JM, DeBoer R, Gebrim LH, Masetti R, Masood S, Menon M, Nakigudde G, Ng’ang’a A, Niyonzima N, Rositch AF, Unger-Saldaña K, Villarreal-Garza C, Dvaladze A, el Saghir NS, Gralow JR, Eniu A (2020). Breast cancer treatment: a phased approach to implementation. Cancer.

