# Supplementary material for: Invasive breast Cancer treatment in Tanzania: landscape assessment to prepare for implementation of standardized treatment guidelines
Source: BMC Cancer. 2021 May 10;21:527. doi: 10.1186/s12885-021-08252-2 (PMC8108449; doi:10.1186/s12885-021-08252-2)
Supplement: Supplementary file 1 — Additional file 1 Supplementary Fig. 1. Eligibility, inclusion, and exclusion criteria of study population: Among 235 patients with suspected breast reviewed during the study period, 164 patients were included. [file 12885_2021_8252_MOESM1_ESM.docx]

**Invasive Breast Cancer Treatment in Tanzania: Landscape Assessment to Prepare for Implementation of Standardized Treatment Guidelines**

Rupali Sood M.D. MPH^1^, Nestory Masalu M.D.^2^, Roisin M. Connolly MB BCh M.D.^3^, Christina A. Chao MSc^1^, Lucas Faustine M.D.^2^, Cosmas Mbulwa M.D.^2^, Benjamin O. Anderson M.D.^4^, Anne F. Rositch PhD MSPH.^1^

^1^ Johns Hopkins Bloomberg School of Public Health, Department of Epidemiology, Baltimore, MD 21205, USA

^2^ Bugando Medical Centre, Mwanza, Tanzania

^3^ Cancer Research @ UCC, College of Medicine and Health, University College Cork, Ireland

^4^ Breast Health Global Initiative, Fred Hutchinson Cancer Research Center, Seattle, WA 98109 USA

**Correspondence To:** Dr. Anne F. Rositch, Department of Epidemiology, Johns Hopkins Bloomberg School of Public Health, 615 N. Wolfe St., Office E6150, Baltimore, MD, 21205, [arositch@jhu.edu](mailto:arositch@jhu.edu), 443-287-5095

**664 patients with a breast concern**

(women, age ≥ 30 years)

**235 charts reviewed of patients suspected to have breast cancer**

**71 for exclusion**

44 no available treatment information

5 breast surgical procedures, not for cancer

5 other types of cancer

4 infections

4 normal breasts

9 benign disease (fibroadenoma, cysts, fibrocystic changes)

**164 patients included**

**113 patients** treated for histopathology confirmed invasive breast cancer

5 no adjuvant therapy

2 chemotherapy alone

18 surgery alone

38 surgery, chemotherapy

16 surgery, hormone therapy

31 surgery, chemotherapy, hormone therapy

2 surgery, chemotherapy, hormone therapy, radiation therapy

1 surgery, chemotherapy, radiation therapy

**Supplementary Figure. 1** Eligibility, inclusion, and exclusion criteria of study population: Among 235 patients with suspected breast reviewed during the study period, 164 patients were included.

**51 patients** (no confirmed diagnosis) treated for suspected breast cancer

1 hormone therapy alone

18 chemotherapy alone

17 surgery alone

2 surgery, chemotherapy

3 surgery, hormone therapy

6 surgery, chemotherapy, hormone therapy

1 surgery, chemotherapy, hormone therapy, radiation therapy

2 chemotherapy, hormone therapy

1 chemotherapy, hormone therapy, radiation therapy

**Supplementary Figure 1 Information:**

- File Name: Supplementary Figure 1
- Format: docx
- Title: Eligibility, inclusion and exclusion criteria of study population
- Description: Among 235 patients with suspected breast reviewed during the study period, 164 patients were included; 113 with confirmed histopathologic breast cancer and 51 with suspected breast cancer (no histopathologic diagnosis).
